# Expression of Interleukin-17 associated with disease progression and liver fibrosis with hepatitis B virus infection: IL-17 in HBV infection

**DOI:** 10.1186/1746-1596-8-40

**Published:** 2013-02-28

**Authors:** Wen-Jun Du, Jun-Hui Zhen, Zhao-Qing Zeng, Zhao-Min Zheng, Yan Xu, Lai-Ying Qin, Shi-Jun Chen

**Affiliations:** 1 Shandong University, School of Medicine, Jinan, China; 2 Department of Liver Diseases, Jinan Infectious Disease Hospital, Shandong University School of Medicine, Jinan, China; 3 Department of Pathology, Shandong University School of Medicine, Jinan, China

**Keywords:** Asymptomatic, Hepatitis B surface antigen Carriers, Interleukin-17, Hepatitis B virus, Chronic hepatitis B, Liver cirrhosis, Primary hepatitis carcinoma, Chronic liver failure, Fibrosis

## Abstract

**Background/objectives:**

As a proinflammatory cytokine, interleukin-17 (IL-17) contributes to the inflammation of many autoimmune diseases. We examined IL-17 levels in serum and tissues from patients with chronic hepatitis B virus infection (HBV), and especially evaluated the role of IL-17 in the pathogenesis and progression of liver fibrosis.

**Materials and methods:**

Whole venous blood was obtained from four patient groups: chronic hepatitis B (CHB, n = 47), liver cirrhosis (LC, n = 49), primary hepatocellular carcinoma (PHC, n = 44), chronic liver failure (CLF, n = 33), and a normal control group (n = 20). HBsAg was positive in all patients. Liver biopsy samples were acquired from asymptomatic HBsAg carriers (ASC, n = 35), CHB (n = 57), and LC (n = 31) patients. We performed ELISA to measure IL-17 levels in serum samples, and used reverse RT-PCR to measure IL-17 mRNA levels in peripheral blood mononuclear cells (PBMC). IL-17 protein expression was detected in liver biopsy tissues by immunohistochemistry.

**Results:**

Compared to normal controls, serum IL-17 protein and mRNA levels were significantly higher in the four infection groups. LC patients exhibited the highest serum IL-17 and PBMC mRNA levels. No significant differences were found between the other three groups. High levels of IL-17 were also observed in tissues from CHB and LC patients, compared to ASC. IL-17 expression was mainly located in the portal area and was positively correlated with inflammation grade and fibrosis stage.

**Conclusions:**

IL-17 expression was found to be increased with increasing degrees of liver fibrosis. This suggests that IL-17 may not only induce the inflammation, but also contribute to disease progression and chronicity.

**Virtual Slides:**

The virtual slide(s) for this article can be found here: http://www.diagnosticpathology.diagnomx.eu/vs/5306959258322482

## Introduction

Hepatitis B is a major cause of liver diseases causing chronic hepatitis B virus (HBV) infection in an estimated 400 million people worldwide [1]. Despite advances in the development of specific antiviral therapy, both acute and chronic HBV infections continue to represent important global health problems. Chronic HBV infection includes three phases: an immune tolerant phase with high HBV DNA and minimal liver dysfunction, an immune active phase with high HBV DNA levels, acute liver inflammation, and tissue damage, and an inactive phase with minimal inflammation and liver fibrosis [2-4]. Therefore, host immune responses against the invading viruses actually mediate the immunopathology and contribute to liver dysfunction and tissue damage [4,5].

T cell-mediated adaptive immune response plays an essential role in chronic HBV infection. Efficient viral clearance requires an effective T cell immune response, and memory CD8^+^ T cell-derived interferon (IFN)-γ down regulates HBV replication [6]. However, T cell-mediated chronic inflammation also results in necro-inflammatory responses during chronic HBV infection [4], and is required for the development of hepatocellular carcinoma (HCC) in HBV transgenic mice [7]. Therefore, T cell-mediated adaptive immunity imposes a “double-edged sword” by reducing levels of HBV at the expense of organ injury.

Interleukin17 (IL-17) is a cytokine produced by a newly defined subset of helper T cells. The IL-17 family of cytokines has been reported to be involved in many immune processes, most notably in inducing and mediating proinflammatory responses. This proinflammatory activity is exemplified by their involvement in pulmonary inflammatory responses.Th17 cells are distinct from Th1 or Th2 cells [8]. Differentiation of mouse Th17 cells is dependent on transforming growth factor-β (TGF-β) and interleukin-6 (IL-6) [9-11]. The factors driving human Th17 differentiation remain controversial, but may involve TGF-β, interleukin-1β (IL-1β), IL-6, and IL-23 (IL-23) [12-15]. IL-17 is upregulated in the lesions of patients with various chronic inflammatory diseases, such as pulmonary infection [16], psoriasis [17], inflammatory bowel disease [18], and rheumatoid arthritis [19,20]. Interestingly, a recent study indicates that anomalous induction of IL-17-expressing CD8^+^ T cells lacking the transcription factor, T-bet, and eomesodermin was found to be associated with a progressive inflammatory and wasting syndrome characterized by multi-organ infiltration of neutrophils during infection by lymphocytic choriomeningitis virus (LCMV) [21]. Therefore, IL-17 may play an immunopathological role, resulting in tissue damage during viral infection.

## Methods

### Patients

Twenty normal healthy people, control subjects, were selected from Jinan Infectious Disease Hospital. A total of 173 patients with HBV who were admitted to our hospital from January 2007 to September 2010 were enrolled. Peripheral blood was collected from 20 normal control subjects (14 males and 6 females, mean age ± standard deviation, 34.00 ± 11.59 years) as well as from the 173 patients, of whom 47 patients were diagnosed with CHB (30 males and 17 females, mean age of 33.41 ± 14.58 years; there were 13 cases of mild CHB, 28 of moderate CHB, and 6 of severe CHB). Forty-nine patients had LC (33 men and 16 women with a mean age of 51.92 ± 11.53 years), and 44 patients were diagnosed with primary hepatic carcinoma (PHC), (30 men and 14 women with a mean age of 54.66 ± 7.46 years). The remaining 33 patients were diagnosed with chronic liver failure, including 22 men and 11 women with a mean age of 46.30 ± 6.79 years. A total of 123 patients infected with HBV who were admitted to our hospital from January 2004 to September 2010 were enrolled in the pathological study. Liver tissue and the peripheral blood were collected from 35 ASC (16 men and 9 women with a mean of 33.34 ± 9.75 years), 57 patients diagnosed with CHB, (41 men and 16 women with a mean age of 39.72 ± 13.12 years), and 31 patients diagnosed with LC (20 men and 11 women with a mean age of 45.38 ± 14.59 years). All the patients with CLF, LC, and PHC were positive for the HBV surface antigen (HBsAg). In this study, the clinical diagnosis was based on serum liver tests, hepatitis virus markers, autoantibodies, tumor markers, ultrasonography, and liver histopathology. Values of TBIL greater than 171 μM and PTA less than 40% were used to diagnose liver failure (LF). All cases were stratified into 10 groups (G0 to G4, S1 to S5) according to the modified criteria for grading and staging of chronic hepatitis according to the Hepatitis Consensus Meeting held in China in 2000. All patients were confirmed by the criteria of the Program for Prevention and Treatment of Viral Hepatitis (2000, Xi’an). None of the patients took antiviral or immunomodulatory agents three months prior to the examination or took liver protective agents two months prior to the examination. No patients had other kinds of viral hepatitis, or other conditions such as alcoholic hepatitis, autoimmune diseases, or severe diseases of other organ systems. The study protocol was reviewed and approved by the Jinan Infectious Disease Hospital Ethics Committee, and written informed consent was obtained for all participants.

### Reagents

Human IL-17 Quantikine ELISA kits (D1700) were purchased from R&D Systems (Minneapolis, MN). Kits for human type IV collagen (Col IV), human laminin (LN), human hyaluronic acid (HA), and human N-terminal procollagen III propeptide (PIIINP) were purchased from the Chinese Academy of Sciences, Shanghai Institute of Cell Biology. Lymphocyte separation medium was purchased from the Tianjin Hao Yang Biological Products Corporation. Trizol reagent was purchased from Invitrogen (Carlsbad, CA). M-MuLV reverse transcriptase enzyme was purchased from the Promega Corporation (Madison, WI). Taq enzyme, RNA enzyme inhibitors, olig (dT), and dNTP were all purchased from Takara Bio, Inc. Primers were synthesized by the Shanghai Boya Biotechnology Corporation. Rabbit anti-human IL-17 polyclonal antibody was purchased from Santa Cruz Biotechnology. Goat anti-rabbit IgG polymer and DAB chromogen solution were purchased from the Zhongshan Jinqiao Biotechnology Development Corporation.

### ELISA

Peripheral venous blood was collected from subjects and normal controls. The serum levels of IL-17, and the serum fibrosis markers (serum collagen IV, serum LN, serum HA, and serum PIIINP) were analyzed by a double-antibody sandwich ELISA using specific ELISA kits (Quantikine R&D Systems) according to the manufacturer’s instructions. All serum samples were stored at -70°C until used for analysis.

### Real-time PCR (RT-PCR)

Peripheral venous blood was collected from patients and normal controls. Total RNA was prepared from PBMC using Trizol according to the vendor’s protocol. For the measurement of IL-17A mRNA levels, the following primers were used: forward and reverse primers of human IL-17, 5^′^- AGA GAT ATC CCT CTG TG ATC and 5^′^TAC CCC AAA GTT ATC TCA GG-3^′^; forward and reverse primers for human β-actin5^′^-CATGTACGTTGCTATCCAGGC-3^′^ and 5^′^-CTCCTTAATGTCACGCACGAT-3^′^. The annealing temperature was 55°C.

### Histological assessment, immunohistochemistry, and scoring

All biopsy specimen tissues were fixed in 10% formalin, and processed using a routine protocol which included dehydration and paraffin-embedding.

Samples were stained with hematoxylin and eosin (HE). Fibrosis staging (S) and inflammatory activity grading (G) were scored according to Precautionary and Curative Measures of Hepatitis Virus (PCMHV) criteria. Fibrosis staging was divided into S1-S5 (S1: no fibrosis; S2: portal fibrosis without septa, but with minimal fibrosis of hepatic lobules; S3: per portal fibrosis with few septa and moderate fibrosis of hepatic lobules; S4: septal fibrosis with many septa and structural distortion of hepatic lobules; S5: cirrhosis). Inflammatory activity was divided into G0-G4 (G0 = no histologic necroinflammatory activity, G1 = portal inflammatory activity, G2 = minimal patchy necrosis, G3 = moderate patchy necrosis, G4 = severe patchy necrosis).

Liver biopsies were performed with sure cut needles (16 G × 70-90 mm) following consent of the subjects. The specimens were fixed in formalin, embedded in paraffin, and stained with a streptavidin-peroxidase (SP) immunohistochemical staining method. An intensity score was assigned, representing the average intensity of positive cells (0, none; 1, weak; 2, intermediate; and 3, strong). A proportion score was assigned, which represented the estimated proportion of positive-staining cells (0, <5%; 1, 5%-25%; 2, 26%-%; 3, 51%-75%; and 4, >75%). The proportion and intensity scores were then added to obtain a total score, which ranged from 0 to 8. Slides were scored by pathologists who did not have knowledge of the ligand-binding results or patient outcomes. The total score was further divided into the score as follows: <2, (-); 2-3, (+); 4-5, (++); 6-7, (+++).

#### Measurement of serum liver tests

Alanine aminotransferase (ALT), aspartate aminotransferase (AST), total bilirubin (TBIL), albumin, and globulin were all measured by an automatic biochemical analyzer (CX-7, Beckmann Coulter, Inc.). Prothrombin activity (PTA) was detected by an automatic coagulometer (Compact, STAGO), and HBV-DNA concentrations were detected by a fluorescence quantitative PCR.

### Statistics

All data were analyzed using the statistical package SPSS version 11.5 (SPSS, Chicago, IL). The Kruskal Wallis and Mann-Whitney tests were used to compare the continuous variables. Spearman’s correlation coefficient (r) was used to find correlations. All data were summarized as mean and standard deviations. The results were reported as mean ± SD of the indicated number of experiments. A P <0.05 was considered statistically significant. The Mann-Whitney U test was used to make ordinal comparisons. Significance was accepted at a corrected *p* value of 0.005 (among the five groups) and p value of 0.017 (among the three groups). A P <0.05 was considered statistically significant.

## Results

Average serum IL-17 protein values for the four groups (CHB, LC, PHC, and CLF) were 38.9 ± 11.34 pg/ml, 63.9 ± 18.82 pg/ml, 46.8 ± 14.39 pg/ml, 44.0 ± 3.78 pg/ml, respectively, while the control group value was 28.2 ± 7.78 pg/ml. These serum IL-17 levels were 37.9%, 126%, 63%, and 56% higher in the patients with CHB, LC, PHC, and chronic severe hepatitis compared to normal controls, respectively (CHB, *P*=0.002; all others, *P* < 0.001). Of the four groups, the patients with LC exhibited the highest IL-17 levels. The serum IL-17 protein levels were significantly higher in the patients with LC compared to the ones with CHB and chronic severe hepatitis (*P* < 0.001), and were higher in the patients with LC than the ones with PHC (*P* = 0.001). There were no significant differences between serum IL-17 levels in patients with PHC compared to the ones with CHB (*P* = 0.029), between the patients with CHB or the ones with chronic severe hepatitis (*P* = 0.260), and between the patients with the chronic severe hepatitis or ones with PHC (*P* = 0.435) (Table 1). The average values of PBMC IL17A mRNA in the four groups (CHB, LC, PHC, and CLF) were 0.41 ± 0.14, 0.80 ± 0.17, 0.55 ± 0.13, 0.40 ± 0.09, respectively, while the control group value was 0.05 ± 0.07. These values were significantly higher in all four patients groups compared to the normal group (p < 0.001 for all). Of the four patient groups, the patients with LC exhibited the highest PBMC IL17A mRNA levels. PBMC IL17A mRNA levels in the patients with LC were significantly higher than the patients with CHB, PHC, or severe hepatitis (p < 0.001for all). PBMC IL17A mRNA levels were higher in the patients with PHC than in the ones with CHB and the ones with the CLF (p = 0.001 for both). There was no significant difference between the patients with CHB and the patients with CLF (p = 0.857) (Table 1).

**Table 1:** **IL-17 protein concentration in serum and IL-17 mRNA content in PBMC**$\left(\bar{x}\pm s\right)$

	**Cases**	**IL-17(pg/ml)**	**IL-17A mRNA**
Normal controls	20	28.2 ± 7.78	0.05 ± 0.07
CHB	47	38.9 ± 11.34^*##^	0.41 ± 0.14^**##^
LC	49	63.9 ± 18.82^**^	0.80 ± 0.17^**^
PHC	44	46.8 ± 14.39^**#^	0.55 ± 0.13^**##^
CLF	33	44.0 ± 3.78^**##^	0.40 ± 0.09^**##^

^*^P < 0.005, ^**^P < 0.001 compared with normal control, ^#^*P* < 0.005, ^##^*P* < 0.001 compared with LC.

IL-17 protein levels in the liver tissues from patients with LC were higher than the ones with CHB and with ASC (p < 0.01, for both). IL-17 protein levels in the liver tissue from patients with CHB were higher than the ones with ASC (p < 0.01).

**Figure 1:**
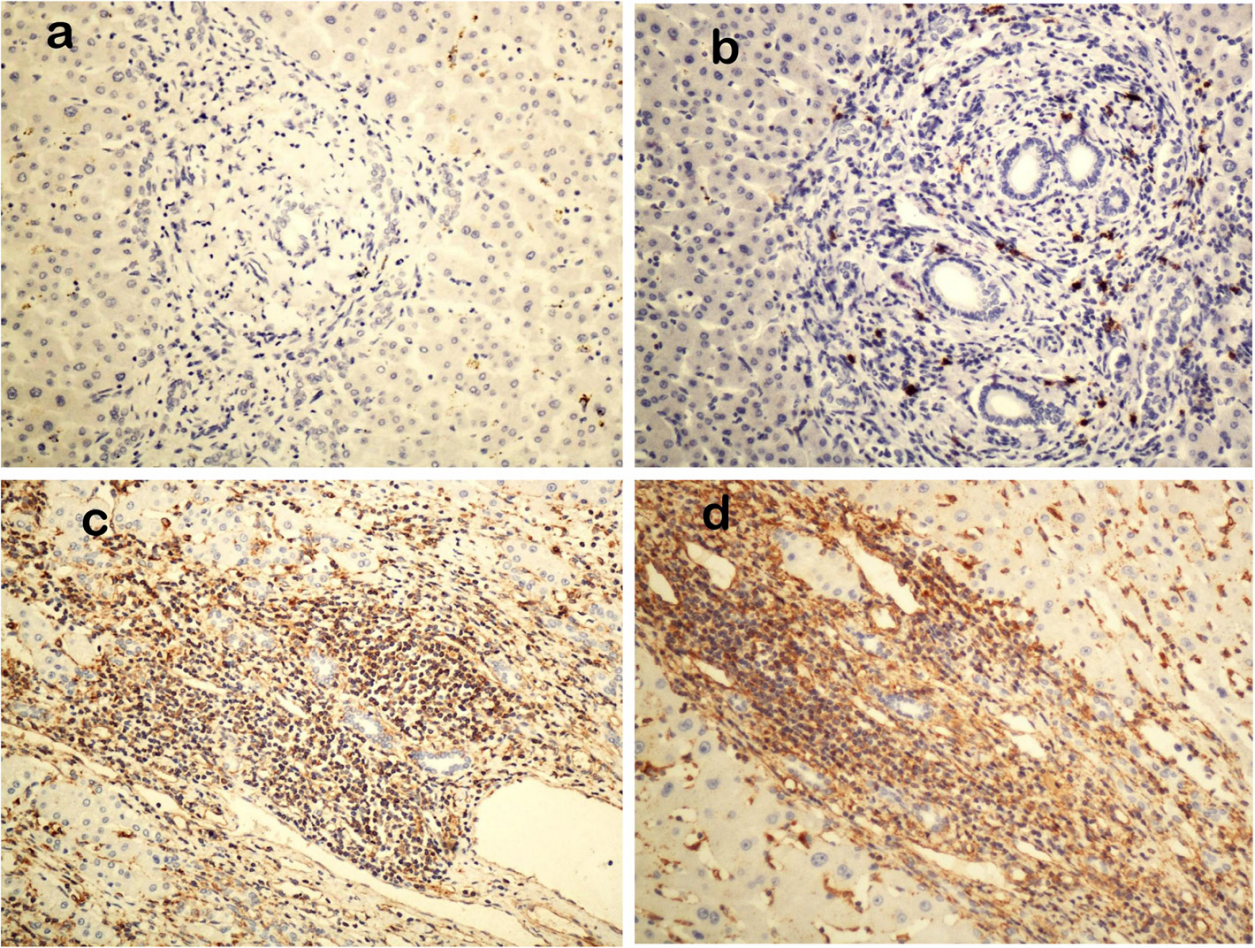
**Immunohistochemical staining for Il-17.** Il-17 protein expression was evaluated by an SP immunohistochemical method. Note that IL-17 was almost absent in normal controls (**a**) and was weak in the liver tissue of ASC patients (**b**). By contrast, moderate and strong staining of IL-17 was identified in CHB (**c**) and LC (**d**), respectively. (×200).

Levels of serum hepatic fibrosis indices in the patients with LC were higher than the ones with CHB (p < 0.01), and those in patients with the CHB were higher than the ones with ASC (p < 0.01). IL-17 protein in the liver tissues was positively correlated with serum collagen IV, LN, and HA (r = 0.883, r = 0.834, r = 0.793, r = 0.722, respectively, and the procollagen III peptide(PIIINP)(r = 0.883, r = 0.834, r = 0.793, r = 0.722, respectively; p < 0.01, for all) (Tables 2, 3, 4). IL-17 expression was mainly localized in the portal area, which was positively correlated with inflammation grade (r = 0.719, p < 0.01) and fibrosis stage (r = 0.692, p < 0.01). Immunohistochemical staining of lymphocytes, fibroblasts, and endothelial cells were positive as seen in Figure 1.

**Table 2:** Serum liver fibrosis markers and liver IL-17 Expression of ASC, CHB, and LC

**Group**	**n**	**Serum collagen IV**	**Serum LN**	**Serum HA**	**Serum procollagen (PIIINP)**	**Liver IL-17 expression**			
						**-**	**+**	**++**	**+++**
ASC	35	109.16 ± 20.17	93.05 ± 11.01	91.96 ± 18.13	8.49 ± 1.77	4	16	10	5
CHB	57	255.33 ± 10.76^*^	104.26 ± 12.73^*^	147.153 ± 16.8^*^	14.60 ± 4.66^*^	2	15	23	17
LC	31	396.71 ± 11. 49^*#^	360.87 ± 10.40^*#^	343.30 ± 16.16^*#^	26.00 ± 6.09^*#^	1	4	9	17

Compared with ASC, *P < 0.001; compared with CHB,#P < 0.001.

**Table 3:** The Relationship between the expression of IL-17 and the degree of liver inflammation

**Group**		**n**		**Expression of IL-17**	
		**-**	**+**	**++**	**+++**
G_0_	5	4	1	0	0
G_1_	31	4	20	7	0
G_2_	44	1	13	23	7
G_3_	36	0	4	11	21
G_4_	7	0	0	2	5

*r* = 0.665, *P* < 0.001.

**Table 4:** The Relationship between the expression of IL-17 and the degree of liver fibrosis

**Group**		**n**		**Expression of IL-17**	
		**-**	**+**	**++**	**+++**
S_1_	10	6	4	0	0
S_2_	27	1	17	9	0
S_3_	42	0	11	22	9
S_4_	21	0	2	8	11
S_5_	23	0	3	5	15

*r* = 0.692, *P* < 0.001.

## Discussion

In this study, we demonstrated that serum IL-17 protein levels and PBMC *IL-17A* mRNA levels were found to be significantly higher in HBV-infected patients when compared to normal controls. IL-17 expression in the liver tissues of the patients was positively correlated with inflammation grade and fibrosis stage, and positively stained lymphocytes suggested that IL-17 takes part in chronic HBV infection. The highest IL-17 levels in the serum and liver were observed in LC patients, suggesting that IL-17 might contribute to the pathogenesis and/or progression of liver fibrosis. Therefore, IL-17 represents a potential therapeutic target for the prevention of liver tissue damage in HBV-infected patients.

Because of the inflammatory reaction of the hepatic tissues in CHB, activated interstitial cells can produce large amounts of TGF-β. TGF-β plays an important role in the differentiation of IL-17. TGF-β together with IL-6 can mediate the *de novo* differentiation of IL-17-producing T cells from naive CD4+ T cell precursors [8]. Th17 is a recently described CD4+ helper T cell subset that produces pro-inflammatory mediators IL-17 and IL-6, which can exacerbate liver damage during chronic HBV infection. One study has also found that peripheral Th17 cells from CHB patients have little capacity to produce IL-22, a cytokine which has been demonstrated to protect against T-cell-mediated hepatitis. The loss of TH17- cells producing IL-22 might exacerbate liver injury in CHB patients [22,23]. IL-17R is expressed in a variety of cell types, which bind the proinflammatory mediator IL-17, and can induce NF-kB activity, improve the induction of NF-kB DNA binding activity, and promote the production of a variety of proinflammatory cytokines by different cell types [9,10]. IL-17 acts synergistically with other pro-inflammatory cytokines in the amplification of the inflammatory response [11].

In the current study, we also found that the serum IL-17 protein levels, PBMC IL-17A mRNA levels, and IL-17 gene expression in the liver were all higher in the patients with LC. IL-17 expression was mainly localized in the portal area and positively correlated with the serum hepatic fibrosis indices (r = 0.692, P < 0.01), which were closely correlated with fibrosis in the liver. We also found that fibroblasts in the liver were positively stained. Liver fibrosis is an important pathological process in the development of liver cirrhosis, which suggests that IL-17 might play an important role in the fibrogenesis and progression of chronic hepatitis B. Some reports have demonstrated that the cytokines TGF-β, interleukin-6, interleukin-1, and TNF-α play important parts in the pathogenesis of liver fibrosis and cirrhosis in CHB [12,13]. IL-17 is biologically closely correlated with some cytokines. TGF-β, together with DC-derived IL-6, is essential for *de novo* differentiation of IL-17-producing T cells from naive CD4 T cells *in vitro*, a process that is amplified by IL-1β and TNFα [8]. Th17 has been shown to induce the secretion of IL-6.

Hepatic stellate cell activation remains the major step in the pathway of fibrogenesis within the liver when inflammation is present. There is some evidence that the activation of the quiescent hepatic stellate cells into an activated myofibroblast phenotype results in the production of fibrillar collagen [24,25]. IL-17 has been found to be highly expressed in many fibrotic-diseases. Whether the effect of IL-17 in the fibrotic process is related with the stellate cell requires further investigation. In our study, we found IL-17 level positively related with the liver fibrosis, the higher fibrosis grade, the higher IL-17 level, but no correlation with PHC and HCC.

In the current study, we found that the serum IL-17 protein levels and the PBMC IL17A mRNA levels were high in the patients with PHC, which suggested that IL-17 takes part in the pathogenesis of PHC. One previous study showed that IL-17 had significant tumor-promoting effects via potentiation of tumor angiogenesis [19]. Another study showed that IL-17 can inhibit the pathogenesis of tumors via an immune-mediated tumor rejection. Therefore, IL-17, like other cytokines, appears to be a pleiotropic cytokine with possible protumor or antitumor effects, in which the predominant effect often depends on the immunogenicity of the tumors [20]. The relationship between IL-17, microvessel density of hepatocellular carcinoma, and the effect of IL-17 in the pathogenesis of the PHC requires further investigation.

The serum IL-17 protein levels and the IL-17 expression in the liver tissue were negatively correlated with albumin levels, and the ratio of albumin to globulin suggested that IL-17 is, to a certain extent, positively correlated with liver damage. ALT can be easily affected by drugs which decrease the enzyme, which may explain why the hepatic histological inflammation grades in some of the patients were not positively correlated with the ALT. The measurement of prothrombin activity is affected by the normal control serum. HBV can induce inflammation of the liver by stimulating the immune system without directly inducing the pathological changes of the hepatic cell. We suspect that this might explain the finding of a lack of relationship between the serum ALT, PTA, HBVDNA concentrations, and the IL-17 in the current study.

Taken together, we have demonstrated that the higher the degree of liver fibrosis, the higher the levels of IL-17 expression. This suggests that IL-17 perhaps may prompt stellate cell and fibroblast proliferation, which may determine the degree of fibrosis. It may also be a valuable indicator for disease progression and prognosis. Increased expression of IL-17 in HBV infected patients also supports a role for IL-17 in the infection, but the exact mechanism of action needs further investigation.

## Abbreviations

IL-17: Interleukin-17;HBV: Hepatitis B virus;ASC: Asymptomatic HBsAg carriers;CHB: Chronic hepatitis B;LC: Liver cirrhosis;LF: Liver failure;PBMC: Peripheral blood mononuclear cells;HCC: Hepatocellular carcinoma;PHC: Primary hepatic carcinoma;IFN: Interferon;TGF-β: Transforming growth factor-β;LCMV: Lymphocytic choriomeningitis virus;Col IV: Human type IV collagen;LN: Human laminin;HA: Human hyaluronic acid;PIIINP: Human N-terminal procollagen III propeptide;ALT: Alanine aminotransferase;AST: Aspartate aminotransferase;TBIL: Total bilirubin;PTA: Prothrombin activity

## Competing interest

None of the authors has an affiliation or conflict of interest.

## Authors’ contributions

Study concept and design: S-JC. Acquisition of data: WJD, J-HZ. Analysis and interpretation of data: W-JD, J-HZ. Drafting of the manuscript: W-JD, J-HZ, S-JC. Critical revision of the manuscript for important intellectual content: S-JC. Statistical analysis: W-JD, J-HZ. Obtained funding: NA. Administrative, technical, or material support: W-JD, J-HZ, Z-QZ, Z-MZ, YX, L-YQ. Study supervision: S-JC. All authors read and approved the final manuscript.
